# Artificial Intelligence in Operating Room Management

**DOI:** 10.1007/s10916-024-02038-2

**Published:** 2024-02-14

**Authors:** Valentina Bellini, Michele Russo, Tania Domenichetti, Matteo Panizzi, Simone Allai, Elena Giovanna Bignami

**Affiliations:** Anesthesiology, Intensive Care and Pain Medicine Division, Department of Medicine and Surgery, University of Parma, Parma, 43126 Italy

**Keywords:** Artificial intelligence, Machine learning, Operating room, Management, Perioperative

## Abstract

This systematic review examines the recent use of artificial intelligence, particularly machine learning, in the management of operating rooms. A total of 22 selected studies from February 2019 to September 2023 are analyzed. The review emphasizes the significant impact of AI on predicting surgical case durations, optimizing post-anesthesia care unit resource allocation, and detecting surgical case cancellations. Machine learning algorithms such as XGBoost, random forest, and neural networks have demonstrated their effectiveness in improving prediction accuracy and resource utilization. However, challenges such as data access and privacy concerns are acknowledged. The review highlights the evolving nature of artificial intelligence in perioperative medicine research and the need for continued innovation to harness artificial intelligence’s transformative potential for healthcare administrators, practitioners, and patients. Ultimately, artificial intelligence integration in operative room management promises to enhance healthcare efficiency and patient outcomes.

## Introduction

The operating room (OR) is healthcare’s epicenter, efficient OR resource management, personnel, equipment, is vital for top-tier surgical care [1]. Recently, Artificial Intelligence (AI) and Machine Learning (ML) integration are transforming OR management, redefining surgical planning and optimization [2]. The journey towards AI and ML in OR management began with a realization: healthcare’s data held untapped potential from patient demographics to surgery histories, anesthesia protocols to recovery room dynamics [3]. In 2015, research on ML in medicine grew exponentially, transitioning from theory to real-world applications [4]. With increased ML understanding and computing power, healthcare is using this technology to tackle complex challenges [5]. In the era of data-driven healthcare, ML became a cornerstone for OR tasks, predicting surgical durations, optimizing schedules, and improving resource use [6]. ML algorithms, like decision trees and random forests, redefined OR efficiency, promising more accurate predictions and proactive decision-making [7]. This systematic review updates our prior work, “Artificial Intelligence: A New Tool in Operating Room Management. Role of Machine Learning Models in Operating Room Optimization” focusing on Feb 2019 to Sep 28, 2023 [4]. In the prior review, we explored ML’s pivotal role in reshaping OR management, emphasizing AI-driven algorithms’ potential for scheduling, case duration prediction, and resource allocation streamlining. In this update, we delve into the latest ML developments in perioperative medicine, exploring how they redefine OR efficiency and patient care. We explore ML’s expansion into perioperative medicine, from Post Anesthesia Care Unit (PACU) resource allocation to reducing surgical case cancellations. We’ll also spotlight integration challenges and opportunities as we aim to maximize AI’s potential for all in healthcare.

## Methods

### Search Strategy

This comprehensive update was conducted in accordance with the Preferred Reporting Items for Systematic Reviews and Meta-Analyses (PRISMA) guidelines. A systematic search was performed across multiple databases, including PubMed, EMBASE, and Scopus databases from February 2019 to September 28, 2023. The search string was adapted from the previous review and comprised various combinations of the following terms: “machine learning,” “anesthesia,” “perioperative,” “PACU,” “operating room,” “recovery room,” and “robotic assisted surgery.”

### Inclusion and Exclusion Criteria

We considered all relevant studies that employed ML techniques in the context of OR, anesthesia, Recovery Room (RR), and PACU management. Studies published before February 2019, abstracts and those not written in English were excluded. Additionally, pediatric and veterinary studies were excluded from the analysis.

### Screening and Selection

Two independent reviewers conducted the screening process in two stages: title/abstract screening and full-text screening. Any discrepancies or uncertainties were resolved through discussion and consensus. After removing duplicates, an initial screening process excluded reviews and conference papers, resulting in a refined pool of potential studies. The remaining full-text articles were assessed, and studies not directly related to ML application were excluded. The final selection included studies published between February 2019 and September 28, 2023, that met the eligibility criteria.

### Data Extraction

Data extraction followed a structured approach, with a focus on study characteristics related to ML methods, patient populations, trial settings, variables, and outcomes. The extracted data were synthesized narratively, focusing on the key themes and findings related to the role of new technologies in perioperative management from an administrative and managerial standpoint. The findings were summarized and presented in a comprehensive manner.

## Results

**Fig. 1:**
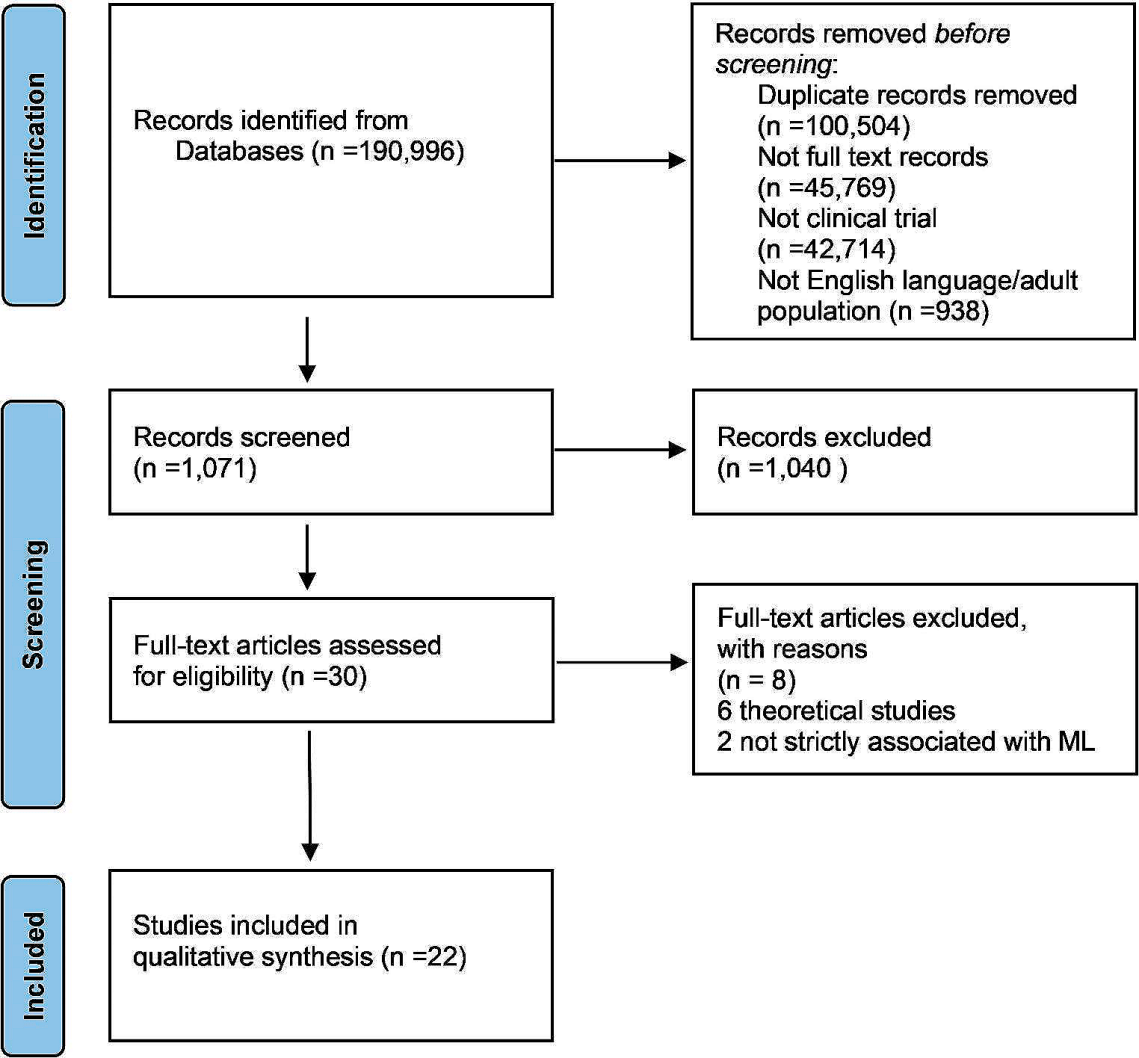
Literature search flow diagram based on PRISMA.

The search returned 90,492 papers published between Feb 2019 and Sep 28, 2023, without duplicates; 44,723 were full text. Only 2,009 were clinical trials and randomized controlled trials. We further skimmed, keeping only English studies involving the adult human population (18 + years), totaling 1,071 studies. After screening the remaining 30 studies, we discarded eight papers: two were not strictly related to ML application, and six were theoretical studies. In the final selection, 22 studies were included in the analysis [8–29]. Figure 1 displays the PRISMA flowchart.

Tables 1 and 2, and 3 summarize key study characteristics, focusing on ML methods, populations, trial settings, variables, and outcomes. Table 1 predicts surgical intervention duration, Table 2 covers PACU stay prediction, and Table 3 focuses on surgical procedure cancellations.

**Table 1:** Main studies about prediction of surgical time

Author, year	Country	Study design	Type of procedure	Main outcomes	Objective	Final Cohort	Type of AI	Prediction Performance	External validation
Bartek MA. J Am Coll Surg. 2019 Oct.	USA	Monocentric, retrospective, observational study	All surgeries	Surgical time prediction	Development of statistical models to improve estimation of case-time duration.	14 345 cases	Random forest and XGBoost	The ability to predict cases within 10% improved from 32% using our institutional standard to 39% with the ML surgeon-specific model. Models with accuracy greater than or equal to that of schedulers (i.e. >75%) constituted 45% of all models. These models were notably superior to the surgeon schedulers with within 10% prediction as high as 50% compared to 32%.	No
Martinez O. Comput Methods Programs Biomed. 2021 Sep.	Colombia	Monocentric, observational study	Single procedures surgeries	Surgical time prediction	Optimization of the OR efficiency by improving the surgery scheduling task, which requires the estimation of surgical time duration.	81 248 cases	Linear Regression, Regression Trees, Support Vector Regression and Bagging Regression Trees	The best overall performance was obtained using Bagged Trees (26 min RMSE, 3.16 min training time, 0.49 min testing time) when using a subset of the DB with the nine specialties containing 80% of the surgeries. Bagged Trees also outperformed the experience-based method with a lower RMSE.	No
Jiao Y. Br J Anaesth. 2022 May.	USA	Multicentric, retrospective, observational study	All surgeries	Methods to predict procedure duration	Development of a machine learning approach that continuously incorporates preoperative and intraoperative information for forecasting surgical duration.	70 826 cases	Modular artificial neural network	The modular artificial neural network had the lowest time error (CRPS mean = 13.8; standard deviation = 35.4 min), which was significantly better (mean difference = 6.4 min [95% confidence interval: 6.3–6.5]; *P* < 0.001) than the Bayesian approach. The modular artificial neural network also had the highest accuracy in identifying operating theatres that would overrun 15:00 (accuracy at 1 h prior = 89%) compared with the Bayesian approach (80%) and a naive approach using the scheduled duration (78%).	Yes
Hassanzadeh H. BMC Med Inform Decis Mak. 2022 Jun.	Australia	Monocentric, observational study	Elective and emergency surgeries	Predicting daily surgery demand and by medical specialty	Utilization of operating theatre data to provide decision support for improved theatre management.	99 732 surgeries on 63 697 unique patients.	Rolling window, Regression (Linear), Regression (Poisson), Regression (Negative binomial), Decision tree, Random forest, SVM (Linear, RBF, Sigmoid, Poly, Bagging regressor, Gradient boosting regressor, XGBoost regressor, Ensemble regressor	Predicting operating theatre demand is a viable component in theatre management, enabling hospitals to provide services as efficiently and effectively as possible to obtain the best health outcomes. They could be predicted with 90% accuracy.	No
Abbou B. Big Data Cogn Comput. 2022 Jul.	Israel	Two-centre, observational, retrospective study	All surgeries	Expected length of stay	Improvement of the productivity and utility of operating rooms.	102 301 cases in hospital 1 and 149 308 case in hospital 2	Naïve model based on the median length of similar surgeries and XGBoost model	Using different measures of performance evaluations, the XGBoost models performed better than the naïve models: the MAE was 21.5 compared to 25.4 in hospital 1 and 25.3 compared to 28.7 in hospital 2; RMSE, 36.6 vs. 49.0 (hospital 1), 40.3 vs. 55.0 (hospital 2); PVE, 66.7 vs. 44.0 (hospital 1), 70. vs. 46.8 (hospital 2); and ML2R, 0.46 vs. 0.53 (hospital 1) and 0.46 vs. 0.49 (hospital 2). In the case of MAPE, differences between the naïve and the ML-based model were minor—35.15 vs. 35.37 in hospital 1 and 35.09 vs. 32.48 in hospital 2 according to hospital performance evaluations.	No
Lam SSW. Healthcare (Basel). 2022 Jun.	Singapore and North Carolina	Two-centre, observational, retrospective study	Colorectal surgeries	Estimation of Surgery Durations	Determination of the performance of current surgery case duration estimations and the use of machine learning models to predict surgery duration across two large tertiary healthcare institutions.	7 585 cases (Center-1) and 3 597 cases (Center-2)	CatBoost	The simple MA-based predictions outperform the scheduled duration provided by the OR schedulers across RMSE, MAE, MAPE and proportion of cases within 80–120% of the scheduled actual duration. In center-1, the Model 5 shows the best performance with RMSE 45.18, MAE 23.986 and MAPE 34.40%. In center-2, Model 5 has the best performance, with 56.11% of its predictions falling within +/−20% of the actual duration. Model 5 prediction accuracy (within +/−20%) is 7.78% higher than that of the MA. Model 5 also has the lowest RMSE, MAE and MAPE at 38.48%, 23.61% and 23.36%, respectively.	No
Gabriel RA. Anesth Analg. 2022 Jul. *	California, USA	Observational, retrospective, single-centre study	Orthopedic and ear, nose, and throat surgeries.	Surgery end time and discharge from recovery room	Development of machine learning models that predicted the following composite outcome: surgery finished by end of operating room block time and patient was discharged by end of recovery room nursing shift.	13 447 surgical procedures	Logistic regression, random forest classifier, support vector classifier, simple feedforward neural network, balanced random forest classifier, and balanced bagging classifier. SMOTE	It has been created a model for each start time (1 pm, 2 pm, 3 pm, or 4 pm) and showed that the ensemble learning approaches had the highest AUC scores. The balanced bagging classifier performed best with F1 score of 0.78, 0.80, 0.82, and 0.82 when predicting our outcome if cases were to start at 1 pm, 2 pm, 3 pm, or 4 pm, respectively.	No
Huang L. J Healthc Eng. 2022 Apr.	China	Monocentric, observational study	All surgeries	Surgical time and anesthesia emergence duration prediction	Creation of a surgery and anesthesia emergence duration-prediction system.	15 754 samples	Perceptron	By combining the surgery duration prediction system with the anesthesia emergence duration prediction system, it has a prediction accuracy > 0.95	No
Chu J. Healthcare (Basel) 2022 Aug.	Taiwan	Retrospective, monocentric, observational study	All surgeries	Surgical time prediction	Construction of prediction models to accurately predict the OR room usage time and compare the performance of different models.	124 528 entries of room	XGBoost, Random Forest, Artificial Neural Network, and 1-dimensional Convolution neural network.	They have found the result of their best performing department-specific XGBoost model with the values 31.6 min, 18.71 min, 0.71, 28% and 27% for the metrics of RMSE, MAE, R 2, MAPE and proportion of estimated result within 10% variation, respectively. We have presented each department-specific result with our estimated results between 5- and 10-min deviation would be more informative to the users in the real application.	No
Gabriel RA. JMIR Perioper Med. 2023 Jan	USA	Single-academic-center, retrospective study	Spine surgery	Prediction of case duration	Utilization of an ensemble learning approach that may improve the accuracy of scheduled case duration for spine surgery.	3 189 patients	Multivariable linear regression, Random Forest regressors, bagging regressors, and XGBoost regressors.	The XGBoost regressor performed the best with an explained variance score of 0.778, an R 2 of 0.770, an RMSE of 92.95 min, and an MAE of 44.31 min. Based on SHAP analysis of the XGBoost regression, body mass index, spinal fusions, surgical procedure, and number of spine levels involved were the features with the most impact on the model.	No
Eshghali M. Ann Oper Res. 2023 Jan.	Iran	Observational, single-centre study	All surgeries	Prediction of surgical duration	Development of an approach for scheduling and rescheduling for both elective and emergency patients in OTs.	All cases in the first 20 weeks of 2020	Random Forest, Genetic Algorithm, Particle Swarm Optimization, traffic congestion index, CPLEX.	The results show that by applying the proposed model, the performance of OT can improve by approximately 10.5% on average.	No
Miller LE. Otolaryngol Head Neck Surg. 2023 Feb.	USA	Monocentric, observational study	Otolaryngology surgical cases	Prediction of surgeries duration	Improvement of ML methods by projecting case lengths over existing non-ML techniques for otolaryngology–head and neck surgery cases.	50 888 cases	CatBoost and XGBoost	The CatBoost model demonstrated better predictive ability (RMSE = 38.2, MAE = 23.2) than the XGBoost model (RMSE = 39.3, MAE = 24.3) (*P* = 0.041). However, both performed better than the baseline model (RMSE = 46.3, MAE = 32.8) (*P* < 0.001) reducing operative time MAE by 9.6 min and 8.5 min compared to current methods, respectively.	No
Zhong W. J Clin Monit Comput. 2023 Sep.	USA	Retrospective, monocentric, observational study	Open reduction internal fixation of radius fractures	Prediction of surgeries duration	Demonstration of a proof-of-concept study for predicting case duration by applying natural language processing (NLP) and machine learning that interpret radiology reports for patients undergoing radius fracture repair.	201 cases	Baseline Model, Linear regression, Random Forest regressor, Multilayer perceptron neural network, Performance Metrics, K-Folds Cross-Validation	The average root mean squared error was lowest using feedforward neural networks using outputs from ClinicalBERT (25.6 min, 95% CI: 21.5–29.7), which was significantly (*P* < 0.001) lower than the baseline model (39.3 min, 95% CI: 30.9–47.7). Using the feedforward neural network and ClinicalBERT on the test set, the percentage of accurately predicted cases, which was defined by the actual surgical duration within 15% of the predicted surgical duration, increased from 26.8 to 58.9% (*P* < 0.001).	No
Adams T. Comput Methods Programs Biomed. 2023 Jun.	New Zeland	Retrospective, monocentric, observational study	Surgical operations	Prediction of procedure durations	Two methods for incorporating the medical information about a surgical procedure into the prediction of the duration of the procedure.	35 000 surgical operations	Linear regression	The ontological information provides an improvement in the continuous ranked probability scores of the prediction of procedure durations from 18.4 min to 17.1 min, and from 25.3 to 21.3 min for types of procedures that are not performed very often.	No
Yeo I. Arch Orthop Trauma Surg. 2023 Jun.	USA	Retrospective, monocentric, observational study	Total knee arthroplasty	Prediction of surgeries duration	Development of an accurate predictive model for surgical operative time for patients undergoing primary total knee arthroplasty.	10 021 patients	Artificial Neural Networks, Random Forest and K-Nearest Neighbor.	Younger age (< 45 years), tranexamic acid non-usage, and a high BMI (> 40 kg/m2) were the strongest predictors associated with surgical operative time. The accurate estimation (AUC = 0.82) is important in enhancing OR efficiency and identifying patients at risk for prolonged surgical operative time.	No
Strömblad CT. JAMA Surg. 2021.	USA	Single-center, 2-campus, randomized clinical trial, prospective study	Colorectal and gynecology surgery	Prediction of the duration of each scheduled surgery, measured by (arithmetic) mean (SD) error and mean absolute error.	Assessement of accuracy and real-world outcome from implementation of a machine learning model that predicts surgical case duration.	683 patients	Random Forest	The implementation of a machine learning model significantly improved accuracy in predicting case duration and led to reduced patient wait time, no difference in time between cases (i.e., turnover time or surgeon wait time), and reduced presurgical length of stay compared to the control group. The SD for colorectal service in the intervention arm would have been reduced from 87 to 70 for the mean absolute error SD and 103 to 86 for the mean error SD.	No
Rozario N. Can J Surg. 2020	Canada	Observational, retrospective, single-centre study	All surgeries	Optimization of surgeries time	Creation of customized models to optimize the efficiency of operating room booking times.	10 553 cases	Python programming language combined with the open source OR-Tools software suite from Google AI	The optimized schedule had 113 min of PACU holds [95% CI: 110, 115 min], a 76% reduction; in addiction to that, 26 min of delays occurred [95% CI: 25, 27 min], corresponding to an 80% reduction in PACU admission delay time [95% CI: 79%, 81%].	No

ML: Machine Learning. RMSE: Root Mean Square Error. CRPS: Continuous Ranked Probability Score SVM: support vector machine. MAE: mean absolute error. MAPE: mean absolute percentage error. R2: coefficient of determination. OT: Operating Theatre. AUC: area under the curve. PACU: Post Anesthesia Care Unit.

**Table 2:** Main studies about PACU length of stay

Author, year	Country	Study design	Type of procedure	Main outcomes	Objective	Final Cohort	Type of AI	Prediction Performance	External validation
Schulz EB. Br J Anaesth. 2020.	Queensland, Australia.	Observational, retrospective, single-centre study	All cases involving an anaesthetic doctor	PACU LOS	Production of case-mix and risk-adjusted post anesthesia care unit length of stay (LOS) benchmarks for integration into modern reporting tools.	67 325 cases	MinMax scaling	This predictive model was able to account for much of the variability observed in individual anaesthetists’ mean PACU LOS (Spearman’s r2 = 0.57). By subtracting the predicted PACU LOS, anaesthetists fell in a much tighter range, with 80% of anaesthetists having a mean LOSD that fell in a band of only 4.3 min, compared with a spread of 24 min for unadjusted mean LOS.	No
Cao B. Ann Palliat Med. 2021.	China	Observational, retrospective, monocentric study	Laparoscopic cholecystectomy	PACU LOS	Development of a predictive nomogram to aid in identifying which LC patients are more likely to be subjected to prolonged PACU LOS.	913 patients	LASSO regression model, C-index, calibration plot, and DCA.	This model displayed efficient calibration and moderate discrimination with a C-index of 0.662 (95% confidence interval, 0.603 to 0.721) for the training set, and 0.609 (95% confidence interval, 0.549 to 0.669) for the test set. DCA demonstrated that the prolonged PACU LOS nomogram was reliable for clinical application when an intervention was decided at the possible threshold of 7%.	No
Tully JL. J Med Syst. 2023.	California, USA	Observational, retrospective, monocentric study	Outpatient surgical procedures	PACU LOS	Development of machine learning models to predict ambulatory surgery patients at risk for prolonged PACU length of stay and then to simulate the effectiveness in reducing the need for after-hours PACU staffing.	10 928 Outpatients	Logistic regression, feedforward neural network, XGBoost regressor, balanced random forest classifier, balanced bagging classifier, and Random Forest classifier.	Female sex (*P* < 0.0001) and scheduled surgical case duration (*P* < 0.0001) were associated with prolonged PACU LOS. Based on AUC, the best performing model with SMOTE was XGBoost (AUC 0.779), whereas the worst performing model with SMOTE was logistic regression without SMOTE (AUC 0.718)	No
Gabriel RA. Anesth Analg. 2022 Jul. *	California, USA	Observational, retrospective, monocentric study	Orthopedic and ear, nose, and throat surgeries	Surgery end time and PACU LOS	Development of machine learning models that predicted the following composite outcome: surgery finished by end of operating room block time and patient was discharged by end of recovery room nursing shift.	13 447 surgical procedures	Logistic regression, Random Forest classifier, support vector classifier, simple feedforward neural network, balanced Random Forest classifier, and balanced bagging classifier. SMOTE.	It has been created a model for each start time (1 pm, 2 pm, 3 pm, or 4 pm) and showing that the ensemble learning approaches had the highest AUC scores. The balanced bagging classifier performed best with F1 score of 0.78, 0.80, 0.82, and 0.82 when predicting our outcome if cases were to start at 1 pm, 2 pm, 3 pm, or 4 pm, respectively.	No

PACU: Post Anesthesia Care Unit. LOS: length of stay. AUC: area under the curve. DCA: decision curve analysis.

**Table 3:** Main studies about risk of surgery cancellation

Author, year	Country	Study design	Type of procedure	Main outcomes	Objective	Final Cohort	Type of AI	Prediction Performance	External validation
Zhang F. J Healthc Eng. 2021.	China	Observational, retrospective, monocentric study	Elective urologic surgeries	Risk of surgeries cancellation	Identification of surgeries with high cancellation risk	5 125 cases	Random Forest, logistic regression, XGBoost-tree, support vector machine-linear, and neural networks.	The average AUCs in the test set exceeded 0.65, with the maximum of AUC (0.7199, RF, original sampling, and backward selection strategy).	No
Luo L. Health Informatics J. 2020 Mar.	China	Observational, retrospective, monocentric study	Elective urologic surgeries	Risk of surgeries cancellation	Identification of surgeries with high risks of cancellation	5 125 cases	Random Forest, XGBoost linear and tree, SVM linear and radial.	The optimal performances of the identification models were as follows: sensitivity − 0.615; specificity − 0.957; positive predictive value − 0.454; negative predictive value − 0.904; accuracy − 0.647; and area under the receiver operating characteristic curve − 0.682. The random forest model achieved the best performance.	No

AUC: Area under the Curve. RF: Random Forest. SVM: Support Vector Machine

Among the 22 studies analyzed [8–29], sixteen primarily focused on predicting the duration of surgical cases [8–13, 15–24], three centered on predicting the length of stay in the PACU [25–27]. One study addressed both aspects [14], while only two studies examined the identification of surgical cases at high risk of cancellation [28, 29]. Additionally, it is noteworthy that only one of the studies is a randomized clinical trial [23], suggesting a need for more robust experimental designs in this research domain. In the selected studies, the most frequently used machine learning algorithms are represented by Random Forest, XGBoost, Linear Regression, Support Vector Regression (SVR), Neural Networks, Bagging, Ensemble Methods, Perceptron, CatBoost, and Logistic Regression. All of them [8–29] demonstrated the capability to enhance predictive accuracy for surgical durations, PACU length of stay, and high-risk surgical case cancellation predictions. Notably, XGBoost exhibited the best overall performance when used. Ensemble methods, like Bagging and Random Forest, improved prediction accuracy by combining models [14]. ML models also optimized scheduling and resource allocation. For instance, Hassanzadeh et al. [11] predicted daily operating theatre arrivals with 90% accuracy, optimizing staffing and resource allocation. Several studies, including those from Bartek et al. [8] and Lam et al. [13], emphasized the importance of tailoring ML models to individual surgeons or considering additional patient and surgery-related factors.

**Fig. 2:**
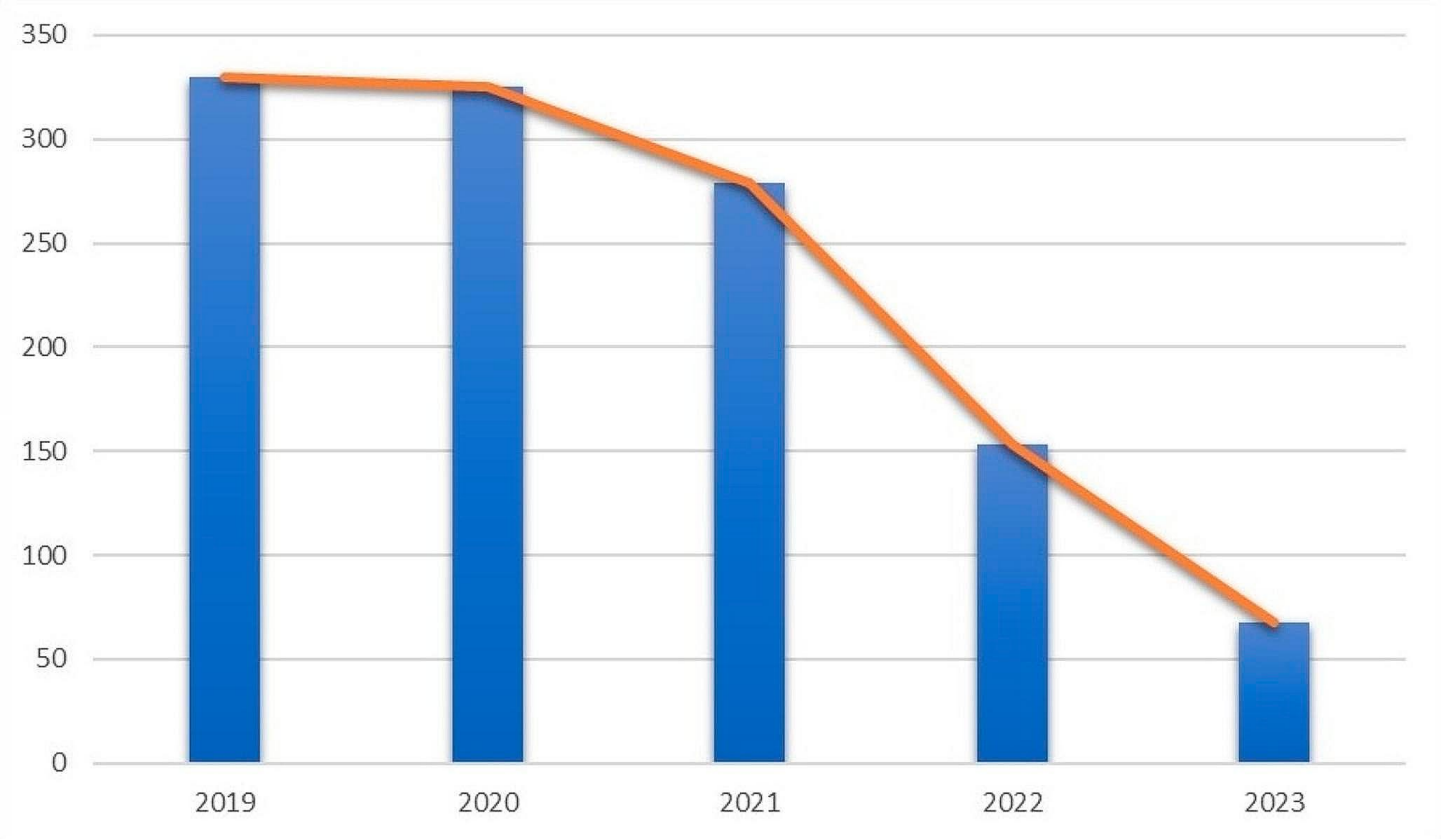
Publication per year since 2019. Note: the timeline counts all publication dates for a citation as supplied by the publisher. These dates may span more than one year. This means the sum of results represented in the timeline may differ from the search results count

The observed trend in scientific paper publications on ML in perioperative medicine showed an increase from 2015 to 2019 [4], followed by a decline (Fig. 2).

**Fig. 3:**
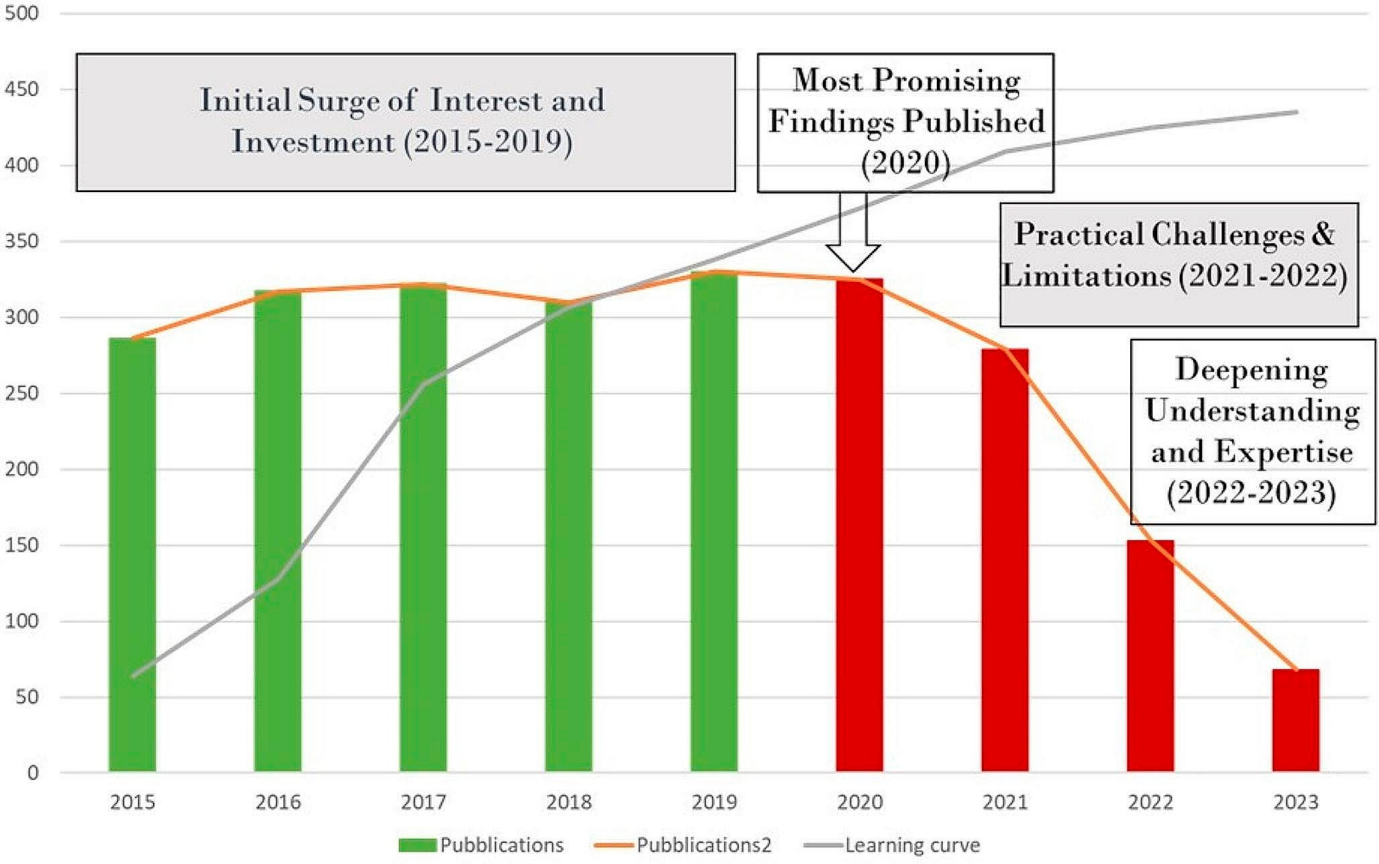
Learning curve of artificial intelligence and publications

This may be indicative of several factors. Initially, there was a surge in interest and investment in ML applications, optimizing OR management, cost reduction, and patient care quality improvement. However, the decrease from 2020 onwards may be due to promising research already being published, practical challenges, or a need for deeper understanding and resources. Characteristic of the learning curve are represented in (Fig. 3).

**Fig. 4:**
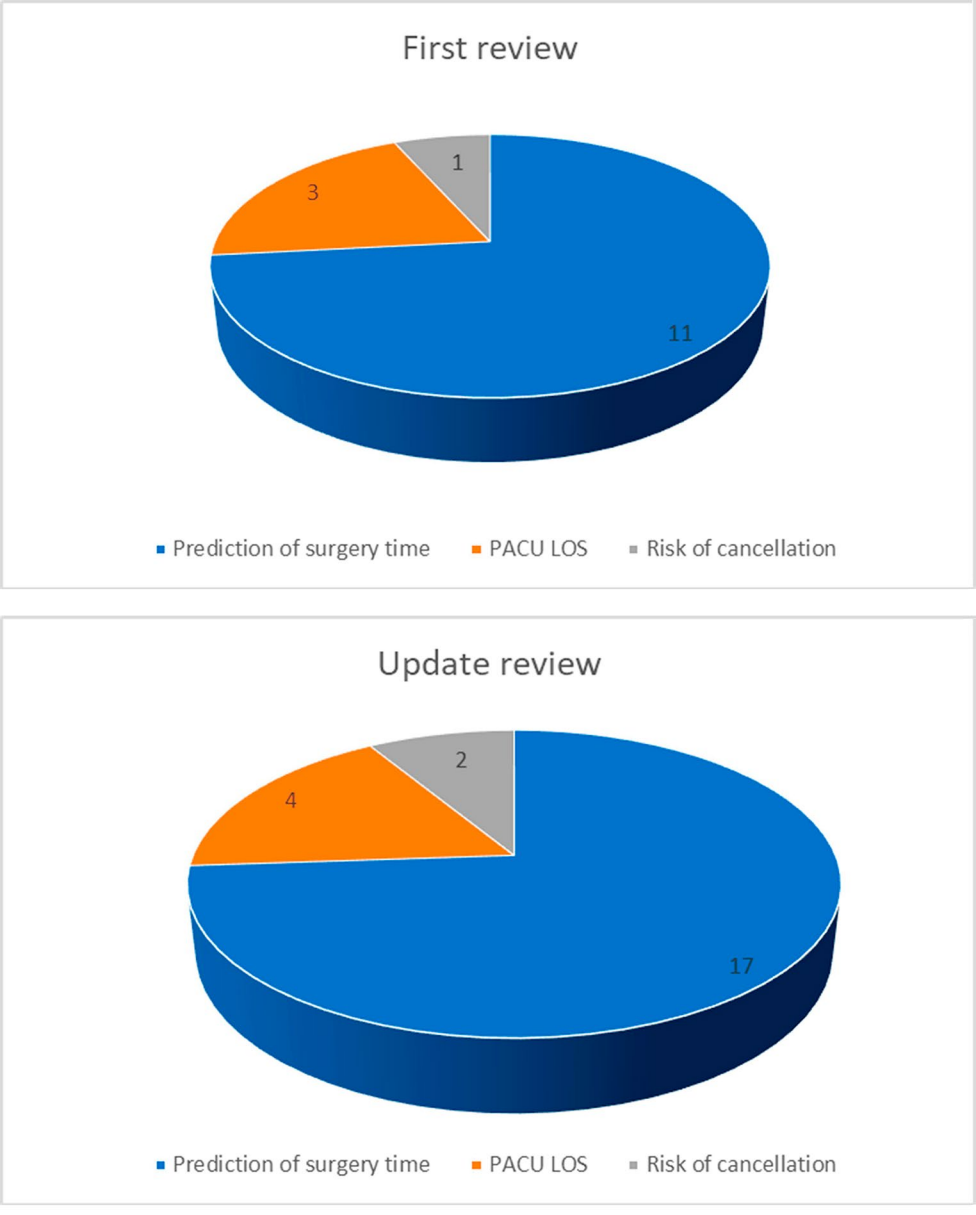
Number of publications per area

This trend reflects the evolving nature of ML in perioperative medicine, necessitating a detailed analysis of research landscape, funding, technology, and evolving priorities. Although studies have demonstrated the effectiveness of AI/ML systems in OR applications, physicians’ hesitancy or reluctance to incorporate these systems into decision making remains a significant barrier. This phenomenon is caused by many factors. The complex nature of AI/ML technologies, particularly in healthcare setting, can contribute to slow adoption in clinical practice. For clinicians, it may be a challenge to understand the algorithms and processes that underpin these systems. The novel and evolving nature of AI technologies may create a perceived risk, causing clinicians to hesitate to fully embrace and trust these tools. Clinicians may not be sufficiently familiar with the concepts and operation of ML/AI systems. Failure to educate and train on how these technologies work can lead to skepticism. Filling this knowledge gap is essential to build trust and confidence among clinicians. Furthermore, in the OR, where patient safety is paramount, clinicians may be particularly reluctant to adopt technologies that may impact patient outcomes. Concerns about the reliability and safety of AI systems may contribute to a conservative approach to their adoption. While studies have demonstrated the efficacy of AI/ML systems in controlled environments, clinicians may be reluctant to consider their applicability and generalisability in the real world. Limited clinical validation and insufficient evidence of improved patient outcomes in diverse scenarios can hinder the acceptance of these technologies. Moreover, clinicians often face ethical and legal considerations when integrating AI into patient care. Issues related to data privacy, liability, and the ethical implications of automated decision-making can contribute to hesitancy in adopting ML/AI systems in ORs. Finally, effective communication and collaboration between data scientists, engineers, and clinicians are crucial. Misalignment in goals, expectations, and language between these interdisciplinary teams can lead to misunderstandings and hinder the successful deployment of AI in clinical settings. Addressing these factors involves not only improving the explainability and transparency of AI models but also implementing robust education and training programs for clinicians [30]. Building a collaborative environment that involves clinicians in the development process, ensuring rigorous clinical validation, and addressing ethical and legal concerns are essential steps toward fostering trust and acceptance. Overcoming these challenges can contribute to accelerating the integration of AI/ML systems in OR decision-making processes. Figure 4 Illustrates a comparison of the number of publications in each area between the previous version of the review and this update.

## Discussion

Out of the 22 selected papers [8–29], 17 focus on predicting the duration of surgical planning [8–24]. This finding underscores the crucial role of accurate estimation in surgical case duration for effective operating room management. It presents a complex and multifaceted challenge that profoundly impacts OR scheduling, resource allocation, and overall operational efficiency. Our previous review [4] primarily highlighted the promising results of a proprietary algorithm known as leap Rail® [31]. While it exhibited an improvement in predictive accuracy compared to traditional methods, our updated review reveals a more nuanced picture. More recent studies, such as the work by Bartek and colleagues [8], have delved deeper into the use of machine learning models, emphasizing the importance of surgeon-specific models. These newer models outperform service-specific ones and significantly enhance the accuracy of case-time predictions, offering substantial benefits in terms of operating room management. Our updated analysis also demonstrates the dominance of XGBoost in machine learning models over other algorithms, including the random forest model and linear regression. XGBoost’s superior predictive capabilities are showcased, which is a notable deviation from the earlier review’s focus on leap Rail® [30]. This underlines the rapid advancements in machine learning technology and its potential to refine surgical case duration predictions. However, is important to keep in mind that different outcomes could require different ML algorithms. [32] Another key finding in the previous review was the potential cost savings associated with accurate surgical case duration predictions in robotic surgery. However, our updated review provides new insights. Jiao and colleagues [11] introduced the use of modular artificial neural networks (MANN) for predicting remaining surgical duration. MANNs are neural networks equipped with external memory. They excel at tasks requiring context and sequential reasoning, making them suitable for certain clinical applications. They leveraged anesthesia records from a diverse range of surgical populations and hospital types, showcasing the robustness and adaptability of their model. MANN consistently outperformed Bayesian statistical approaches, particularly during the last quartile of surgery, indicating its potential for cost savings and operational efficiency improvements. The study also assessed the generalizability and transferability of the MANN model. It found that even healthcare systems with lower operative volumes could benefit from fine-tuning a model trained at larger nearby systems. It also highlighted the lack of meaningful information in the anesthesia record during certain phases of surgery, suggesting room for improvement. This study underscores the rapid advances in machine learning algorithms and their application in real-world surgical scenarios. Variational autoencoders (VAEs), which are generative models designed for learning latent representations of data, also fit in this context. They consist of an encoder and a decoder. The encoder maps input data to a probability distribution in a latent space, and the decoder reconstructs data from samples in this latent space. Linking advanced models like MANNs and VAEs to clinical sense implies that these models could contribute to the field of personalized medicine by learning patient-specific representations, enabling tailored treatment plans and also address clinical needs, enhance diagnostics, improve patient outcomes, or streamline healthcare processes [33]. The work conducted by Strömblad et al. [23], a single-center, randomized clinical trial brought additional insights. They explored the accuracy of predicting surgical case durations using a machine learning model in comparison to the existing scheduling-flow system. This research emphasized the benefits of a comprehensive and data-driven prediction approach, which resulted in a significant reduction in mean absolute error (MAE), contributing to enhanced prediction accuracy. Importantly, this decrease in MAE translated into reduced patient wait times without adversely affecting surgeon wait times or operational efficiency, indicating a harmonious balance between efficiency and patient outcomes. This study is the first and only randomized clinical trial on the subject, to our knowledge, representing a significant milestone.

When comparing the reviews, both the previous [4] and the updated one underscore the potential benefits of improved prediction accuracy in surgical scheduling and operating room management. However, the newer studies provide more specific insights into practical implications. Bartek and colleagues’ work [8] shows a reduction in wait times and resource utilization through the implementation of machine learning-driven models. This has a significant impact on patient outcomes without disrupting operational efficiency, reinforcing the value of these predictive models in real-world healthcare settings. In comparing the updated review of predictive models for PACU length of stay with the previous version [4], we can discern a substantial evolution also in this field. The earlier review had already acknowledged the importance of improving hospital organization and internal logistics to reduce the costs associated with time and space waste in healthcare [4]. It had highlighted issues of congestion in the PACU due to inadequate surgical planning, which often led to patients being held in the OR when PACU beds were unavailable, incurring higher costs. In the current update, we have expanded our analysis to include more recent studies, specifically focusing on predicting PACU length of stay, and their findings are striking. One study conducted by Schulz and colleagues [25] utilized a dataset of 100,511 cases to develop predictive models for PACU length of stay. They considered variables such as patient age, surgical urgency, duration of surgery, and more to create a neural network model. Notably, the study evaluated individual anesthesiologists, categorizing them based on their mean PACU length of stay. The predictive model, relying on routinely collected administrative data, significantly explained variations in individual anesthesiologists’ mean PACU length of stay. This study underscored the practicality of deploying predictive models within existing hospital infrastructure. Tully and colleagues’ research [27], another notable study in this field, aimed to develop a model that could classify patients at high risk for a prolonged PACU stay of ≥ 3 h. The study considered factors like surgical procedure, patient age, and scheduled case duration. The most effective model was XGBoost, which significantly improved the ability to predict prolonged PACU stays. Furthermore, by using the XGBoost model’s predictions, cases were re-sequenced based on the likelihood of a prolonged PACU stay, which led to a substantial reduction in the number of patients in the PACU after hours. These recent studies collectively signify a remarkable shift in the field of PACU length of stay prediction. They highlight the potential of predictive models, machine learning, and data-driven approaches to enhance healthcare quality and operational efficiency. The adoption of big data analytics and optimization of case sequencing have clear implications for improving patient outcomes and resource allocation. It is evident that these models hold significant promise for healthcare institutions, potentially offering considerable cost savings and enhanced patient care. When comparing these recent findings with the previous version of the review, we see a marked advancement in the sophistication of predictive models. The earlier version primarily emphasized the issue of inefficient PACU use and its financial implications, highlighting the potential for cost savings through improved surgical planning. The new studies demonstrate not only the cost-saving potential but also the power of data-driven predictive models, which can significantly enhance the efficiency and effectiveness of healthcare operations.

One of the significant challenges in the healthcare industry is the unexpected cancellation of surgical cases. Surgical cancellations not only disrupt the workflow of healthcare facilities but also pose risks to patient safety and satisfaction [34]. To address this issue and optimize surgical scheduling, ML techniques have emerged as a promising solution for the early detection of potential cancellations. Comparing the updated review with the previous version [4] reveals substantial advancements in this critical aspect of healthcare management. In the earlier review [4], the focus was on the high costs associated with surgical case cancellations, particularly highlighting the cost variation across different types of surgeries. It underscored the need for automatic classification methods to detect high-risk cancellations from large datasets. Furthermore, the review discussed the potential for ML algorithms, specifically random forest, in identifying surgeries at high risk of cancellation, with the promise of optimizing healthcare resource utilization and cost-efficiency. The current review continues to emphasize the significance of addressing surgical case cancellations in healthcare. For example, Luo et al. [28] significantly contribute to the field by leveraging ML to identify high-risk cancellations. Their research focuses on a dataset of elective urologic surgeries, comprising over 5,000 cases, with the aim of identifying surgeries prone to cancellation due to institutional resource- and capacity-related factors. Authors employed three ML algorithms, including random forest, support vector machine, and XGBoost, and evaluated their performance across various metrics. Their findings revealed the suitability of ML models for identifying surgeries at low risk of cancellation, effectively narrowing down the pool of surgeries with higher risk. Moreover, the random forest models displayed good efficacy in distinguishing high-risk surgeries, with an area under the curve (AUC) exceeding 0.6, indicating an interesting result in this context. Different sampling methods allowed for adjustments in model performance, highlighting the trade-offs between sensitivity and specificity. The study concluded that ML models are feasible for identifying surgeries at risk of cancellation. In a subsequent study by Zhang and colleagues [29] from the same center, the focus shifted to providing effective methodologies for recognizing high-risk surgeries prone to cancellation. They also utilized the same dataset but explored a variety of machine learning models, including random forest, logistic regression, XGBoost, support vector machine, and neural networks. The study identified the random forest model as the top-performing algorithm, achieving a high accuracy of 0.8578 and an AUC of 0.7199. Despite the high specificity and negative predictive value, the study acknowledged the need for improving sensitivity and positive predictive value in identifying high-risk cases. In summary, both studies [28, 29] aim to address the challenge of surgical case cancellations in healthcare using machine learning techniques. They highlight the importance of selecting the right machine learning algorithm for this task and acknowledge the need for improving sensitivity and positive predictive value. Both studies [28, 29] acknowledge limitations related to their focus on elective urologic surgeries within a single hospital and suggest the potential for future research to expand to diverse healthcare settings for improved generalizability. Comparing the two reviews, the earlier version [4] emphasized the need for ML algorithms to address surgical case cancellations but did not delve into specific research findings for a lack of studies on the argument. In contrast, in the current version we provide in-depth insights into the suitability of different ML models for identifying high-risk surgeries. Both reviews share a common theme: the critical role of ML techniques in addressing surgical case cancellations to enhance healthcare resource utilization and cost-efficiency.

In summary, the comparison between the two editions of the systematic reviews on the artificial intelligence integration in operative room management highlights a remarkable evolution in each domain. In the case of surgical case duration estimation, the newer review showcases a shift towards machine learning-based models, notably XGBoost, and a heightened focus on surgeon-specific models. This means the realization of machine learning’s potential, promising increased precision in predictions, cost reduction, and enhanced operating room management. Similarly, in the PACU length of stay prediction domain, the updated review underscores the transformative potential of predictive models, emphasizing the value of big data analytics, optimized case sequencing, and risk-adjusted metrics for improving patient outcomes and resource allocation. It acknowledges the challenges of real-world implementation and the need for further validation through prospective studies and collaborative efforts. Overall, the updated review provides deeper insights into the practical applications of these advanced techniques, offering healthcare providers and managers valuable tools to enhance efficiency, reduce costs, and improve patient care. The shift towards center-specific models in healthcare, particularly for organizational aspects, merits in-depth exploration. This trend reflects the growing recognition that customization based on center-specific variables, such as the type of surgeon or anesthetist, can lead to more accurate predictions and better resource allocation. The balance between clinical and organizational applications in these models remains a key consideration. While clinical models focus on patient-specific factors, organizational models, including center-specific ones, primarily address resource optimization, scheduling efficiency, and cost reduction. The choice between center-specific and clinical models ultimately depends on the specific goals and priorities of a healthcare institution. Regarding clinical implementation, it is crucial to investigate how many of these advanced models will progress beyond research to practical application. The shift towards real-world usability is gaining traction, but not all studies provide tools or software for direct application. A critical aspect is the integration of these models into daily work routines. Successful implementation often involves interdisciplinary collaboration between data scientists, healthcare professionals, and administrators. These tools can be used by a range of stakeholders, including surgeons, anesthetists, scheduling teams, and hospital administrators. Different outputs from these models serve varied purposes. For example, clinical models can guide treatment decisions, while organizational models can enhance resource allocation and scheduling efficiency. The extent to which these models are designed for easy integration and use in daily healthcare operations is a key area of investigation, ultimately impacting their practical utility and impact on patient care and healthcare management.

## Limitations

The limitations of this systematic review include the potential for publication bias, as only articles published in English were included. Additionally, the availability of relevant literature may vary across different databases, potentially impacting the comprehensiveness of the review even if, efforts were made to mitigate these limitations by employing a rigorous search strategy and conducting a thorough screening process. Nevertheless, conducting a comprehensive assessment and formulating definitive conclusions regarding the optimal algorithm for predictive models of perioperative complications remains a challenge due to the diverse nature of settings and variations in the algorithms under review. The lack of standardization across studies has impeded our ability to conduct a meta-analysis utilizing both univariate and multivariate random effect models. Furthermore, most studies exhibit a deficiency in external validation of their models. While the use of AUC as an evaluation criterion is practical, it is essential to acknowledge its limitations, particularly in scenarios involving imbalanced datasets within the realm of AI. The significance of ensuring data quality for the successful application of AI extends across various domains, including research, clinical practice, and health system organization. However, achieving datasets of both high quality and quantity necessitates rigorous scrutiny at every stage of the process, spanning from data collection to the selection of ML models and their algorithms.

## Conclusion

In conclusion, this systematic review provides a comprehensive overview of the recent advancements in the application of artificial intelligence, particularly machine learning, in the management of operating rooms. The analysis of the 22 selected studies spanning from February 2019 to September 28, 2023, sheds light on the evolving landscape of AI-driven solutions in perioperative medicine. The review highlights the pivotal role of machine learning in predicting surgical case durations, optimizing resource allocation in the PACU, and detecting surgical case cancellations. These AI-driven models have demonstrated their potential to significantly enhance the efficiency, cost-effectiveness, and safety of surgical procedures. It is evident that machine learning techniques are increasingly integrated into healthcare management to address complex challenges. Furthermore, the review recognizes that the adoption of machine learning in perioperative medicine is not without its challenges. Issues such as data access, privacy concerns, and the need for extensive validation studies pose hurdles to the widespread implementation of AI solutions. The review also suggests that as the field matures, researchers and practitioners must develop a deeper understanding of AI applications, which may lead to a slowdown in new publications as they tackle more complex questions and challenges. Overall, this systematic review underlines the transformative potential of artificial intelligence, particularly machine learning, in reshaping the management of operating rooms. It calls for continued research, collaboration, and innovation to overcome existing challenges and unlock the full benefits of AI for healthcare administrators, practitioners, and most importantly, patients. As we move forward, the integration of AI into operating room management holds the promise of further enhancing healthcare delivery and improving patient outcomes in the years to come.

## Data Availability

No datasets were generated or analysed during the current study.
